# An NMR Metabolomics Approach to Investigate Factors Affecting the Yoghurt Fermentation Process and Quality

**DOI:** 10.3390/metabo10070293

**Published:** 2020-07-17

**Authors:** Alessia Trimigno, Christian Bøge Lyndgaard, Guðrún Anna Atladóttir, Violetta Aru, Søren Balling Engelsen, Line Katrine Harder Clemmensen

**Affiliations:** 1 Chemometrics and Analytical Technology Section, Department of Food Science, University of Copenhagen, Rolighedsvej 26, 1958 Frederiksberg C, Denmark; alessia@food.ku.dk (A.T.); violetta@food.ku.dk (V.A.); se@food.ku.dk (S.B.E.); 2 DTU COMPUTE, Department of Applied Mathematics and Computer Science, Technical University of Denmark, Richard Petersens Plads, 2800 Kgs. Lyngby, Denmark; cbly@dtu.dk (C.B.L.); gudrunanna89@gmail.com (G.A.A.)

**Keywords:** foodomics, food quality, NMR spectroscopy, metabolic profiling

## Abstract

A great number of factors can influence milk fermentation for yoghurt production such as fermentation conditions, starter cultures and milk characteristics. It is important for dairy companies to know the best combinations of these parameters for a controlled fermentation and for the desired qualities of yoghurt. This study investigates the use of a ^1^H-NMR metabolomics approach to monitor the changes in milk during fermentation from time 0 to 24 h, taking samples every hour in the first 8 h and then at the end-point at 24 h. Three different starter cultures (*L. delbrueckii* ssp. *bulgaricus*, *S. thermophilus* and their combination) were used and two different heat treatments (99 or 105 °C) were applied to milk. The results clearly show the breakdown of proteins and lactose as well as the concomitant increase in acetate, lactate and citrate during fermentation. Formate is found at different initial concentrations depending on the heat treatment of the milk and its different time trajectory depends on the starter cultures: *Lactobacillus* cannot produce formate, but needs it for growth, whilst *Streptococcus* is able to produce formate from pyruvate, therefore promoting the symbiotic relationship between the two strains. On the other hand, *Lactobacillus* can hydrolyze milk proteins into amino acids, enriching the quality of the final product. In this way, better insight into the protocooperation of lactic acid bacteria strains and information on the impact of a greater heat treatment in the initial matrix were obtained. The global chemical view on the fermentations provided using NMR is key information for yoghurt producers and companies producing starter cultures.

## 1. Introduction

The production of fermented milk products has been a long, ever-changing process, starting from spontaneous fermentation until modern production employing carefully chosen conditions and starter cultures. The different conditions applied in fermentation have been found to greatly affect the process dynamics and the end-product [1,2,3,4]. Varying milk as raw material (due to season, breed, feeding, etc.), heat treatment, contents of proteins and/or fats, fermentation temperature, type of starter cultures and inoculation rates can yield significantly different end-product quality attributes.

Yoghurt is the classical fermented dairy product in which fresh milk is added to a starter culture to create a thickened gel-style milk product with good mouthfeel character suitable for breakfast and dessert. The final quality of the yoghurt will depend on the milk composition (substrate) [1] and the starter culture applied, typically consisting of a combination of lactic acid bacteria (LAB). LAB have thus been extensively investigated regarding their fermentation performance [5], more specifically their metabolism and resulting exo-metabolites, which are the key factors that determine the texture and aroma of fermented products [6,7], including also health-related components such as soluble polysaccharides and bioactive peptides [5].

In recent years, metabolomics techniques have been employed for the investigation of fermentation using LAB, for example in wine to assess the malolactic fermentation and other metabolisms [8,9], in cheese to investigate ripening [10] or in cereal-based foods to study the functional and sensorial profiles [11]. The application of ^1^H-NMR metabolomics to monitor milk fermentations is particularly useful due to the capability of providing an unbiased and inherently quantitative global chemical overview of the complex milk samples, in a relatively fast manner, due to the limited sample preparation procedures and speed of spectral acquisition [12,13]. In fact, NMR spectroscopy can be of particular help in the investigation of the time dynamics of fermentation [14]. The drawbacks of this technique can be that when employed with real milk fermentation samples, the viscosity characterizing yoghurt samples, generating high sample inhomogeneity, can make smaller molecules behave similarly to large macromolecules, producing very broad spectral lines.

The goal of this study was to develop a pipeline for the investigation of milk fermentation for yoghurt production when different heat treatments of milk and starter cultures are employed. Moreover, we aimed to test the strengths and weaknesses of this analytical protocol and find the points of improvement for future studies.

Two homolactic LAB: *Lactobacillus delbrueckii* ssp. *bulgaricus* (LB) and *Streptococcus thermophilus* (ST) were investigated. The two strains have different metabolic characteristics that make them protocooperate when added together for fermentation. LB is more proteolytic than ST and thus produces more free amino acids whereas ST has the ability to produce formate from pyruvate. Amino acids and formate are both growth factors and the two strains stimulate each other’s growth in the milk media. Two different heat treatments of the milk were also investigated, in order to understand the implication of this perturbation on the growth of the starter cultures: one at 105 °C (HT) and one at 99 °C (LT), both for 30 min. Since intense heat treatment of milk (and milk powder during long term storage) is known to convert lactose into formate through intermediate products [15], it was of particular interest to investigate how heat treatment affects protocooperation.

The analytical pathway described in this study may be helpful for future investigations of yoghurt and other fermentation products.

## 2. Results

**Figure 1:**
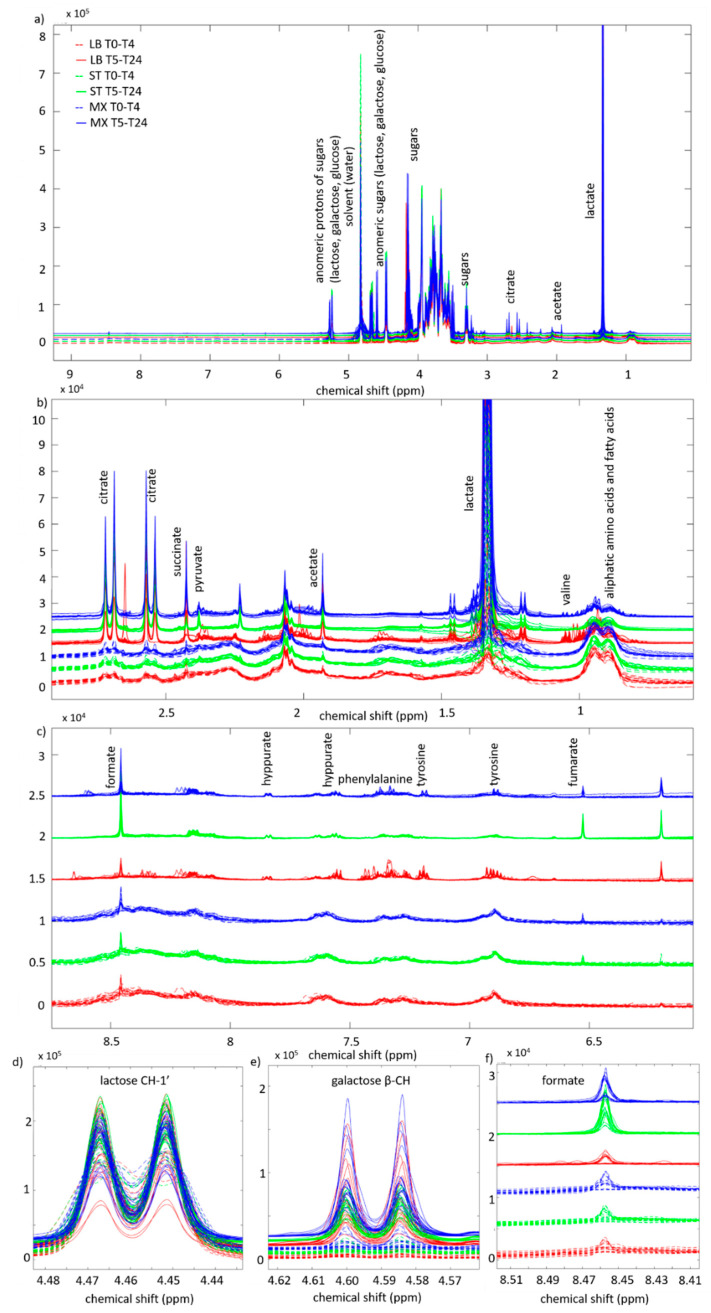
(**a**) Pre-aligned normalized spectra for the first five time-points (T0–T4) and the last five (T5–24) represented respectively with dotted or full lines. Spectra are colored according to strain: LB in red, ST in green and MX in blue. (**b**) Zoomed-in aliphatic (0.6–3 ppm) and (**c**) aromatic (6–9 ppm) regions are also presented, together with some significant signals: lactose (4.44–4.48 ppm) (**d**), galactose (4.57–4.62 ppm) (**e**) and formate (8.41–8.51 ppm) (**f**). The six different spectral sets are represented with a small offset on the vertical axis to better visualize differences.

By visual inspection, the ^1^H-NMR spectra exhibit major differences between the first five time-points (T0–T4) and the last five (T5–T24). As shown in Figure 1, after the first 4 h, large concentrations of deprotonated organic acids are observed (Figure 1b), and broad protein and peptide signals turn into sharper amino acid peaks (Figure 1c). This behavior is the result of fermentation kinetics with a slow growth in the first 4 h, followed by an exponential transition between 4 h and 5 h, to a more stationary phase from 5 h to 24 h.

The presence of such large spectral differences among samples from the same fermentation batch makes the comparison of the two phases (before and after 5 h of fermentation) challenging, because peaks at the same chemical shift represent different chemical compounds in the two phases. Many broad peaks, possibly attributed to proteins, can be observed at the beginning of fermentation and may hinder the analysis of the free amino acids. In fact, these regions could have similar total areas before and after fermentation, although the actual sample composition would be very different, with the latter having larger peptides and protein from milk [16] broken down by the starter cultures into amino acids. Therefore, this information was kept in mind when investigating the metabolite kinetics.

**Figure 2:**
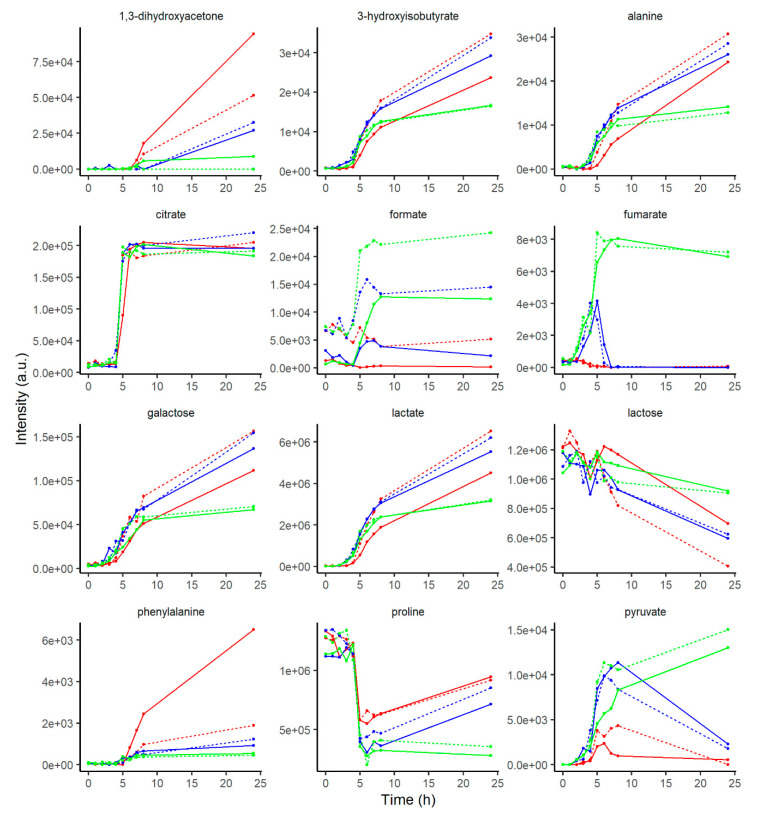
Metabolite development in yoghurt according to the applied experimental design. Points are an average of two replicate samples. Colors denote starter culture *L. delbrueckii* ssp. *bulgaricus* (red), *S. thermophilus* (green) and their combination (blue). The line type denotes milk heating temperature: 99 °C (^___^) and 105 °C (---). e = 10ˆ.

The metabolite concentration development as a function of heat treatment and starter cultures is shown in Figure 2. The concentrations of alanine, galactose and lactate increase during fermentation. Alanine and phenylalanine concentrations after 24 h (stationary phase) are lowest for fermentation with ST, implying that ST has less proteolytic activity. Lactose concentration decreases over time as it is used as substrates for LABs. Formate is found higher in HT than LT milk, and it gradually decreases when only LB is inoculated, whilst it increases when ST is instead employed. Citrate displays a greater increment in concentration between 4 and 6 h of fermentation, with similar development across starters and heat treatments. Pyruvate displays a peculiar behavior, increasing particularly when ST is included as a strain, and when ST is inoculated singularly, pyruvate concentration keeps increasing during fermentation, whilst it decreases when LB is also present. Fumarate shows initially a similar behavior to pyruvate, increasing rapidly when ST is present, though when LB is used also as a strain it is also rapidly used.

**Figure 3:**
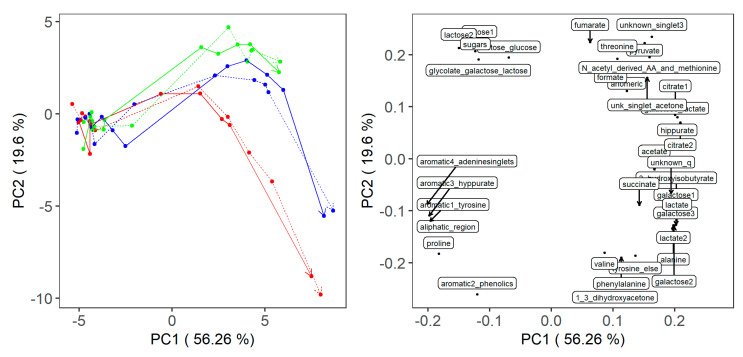
Principal component analysis of selected metabolite concentrations. Left: score plot with fermentation time denoted by trajectories where the arrows mark the 24 h samples. Starter cultures are colored as follows: *L. delbrueckii* ssp. *bulgaricus* red, *S. thermophilus* green and their combination in blue. Milk treated at 99 °C (LT) is represented with a full line, while milk treated at 105 °C (HT) is represented with a dotted line. Right: loading plot. Arrows link the loading plot point to the interval name when excessive clustering is present.

**Figure A1:**
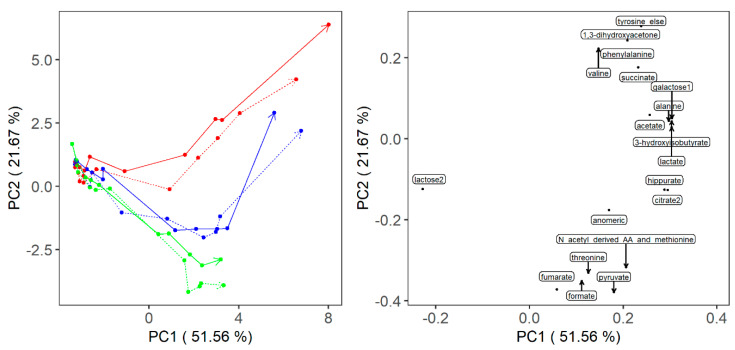
Principal component analysis of 19 signals from known metabolites. Left: score plot with fermentation time denoted by trajectories. Starter cultures are colored as follows: *L. delbrueckii* ssp. *bulgaricus* red, *S. thermophilus* green and their combination in blue. LT milk is represented with a full line, while HT milk with a dotted line. Right: loading plot.

Principal component analysis (PCA) was performed on the mean-centered and unit-scaled metabolite table of peak areas. Figure 3 shows the PCA score and loading plots of PC1 vs. PC2. As it can be observed, the time-points are quite well separated across the first PC, with trajectories for both milk type and strain closely following each other. This is mostly due to differences in sugars and deprotonated organic acids. At the beginning of fermentation, samples are characterized by lactose, whilst proceeding with fermentation amino acids and deprotonated organic acids appear. Moreover, along the second PC it can be seen that the difference between ST and LB is due to a higher concentration of deprotonated organic acids such as fumarate, pyruvate and formate in ST and greater concentrations of amino acids such as phenylalanine or valine in LB. When PCA is calculated using only 19 signals from the known identified metabolites, this becomes even clearer (Figure A1), and a separation between the two milk heat treatments for each strain is also visible.

A heatmap hierarchical clustering analysis was performed on the mean-centered and unit-scaled metabolite table of peak areas. Pearson’s correlation coefficient was used as distance measure among samples to focus on the pattern of sample concentration profiles rather than their magnitudes (Euclidean distance). Average linkage was used to link groups of samples, as average linkage provides more balanced dendrograms compared to single linkage [17]. The gap statistic [18] was used to choose the number of non-random clusters as described in the Materials and Methods section.

**Figure 4:**
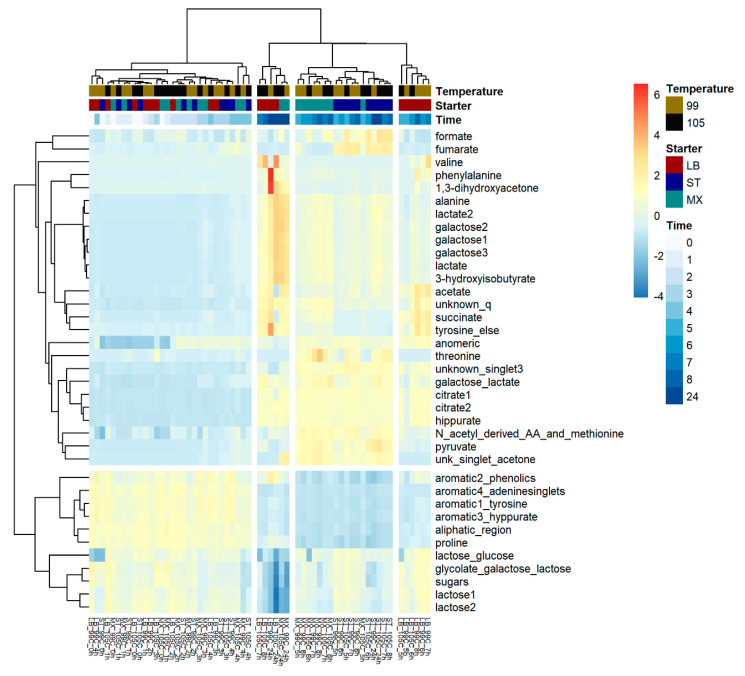
Illustration of hierarchical heatmap clustering of the 37 selected intervals during the fermentation. The experimental design has been emphasized with color-coding. The relative concentration of metabolites is shown with a color gradient from blue (low) to red (high). Spacing in the heatmap shows the significant clusters found according to the gap statistic.

Inspection of the sample dendrogram (Figure 4, top) shows that the samples are branched into two overall clusters, namely the samples before 5 h (Figure 4, heatmap left) and the samples 5 h or more (Figure 4, heatmap right) after inoculation. This is consistent with time being the most important design factor and the exponential growth phase being approximately 4–5 h after inoculation. The metabolite dendrogram (Figure 4, left) shows an initial branching into a cluster of fermentation products, alanine, lactate, acetic acid and citrate (Figure 4, heatmap top), and a cluster of fermentation substrates such as lactose and the aromatic region (Figure 4, heatmap bottom).

## 3. Discussion

### 3.1. Fermentation Kinetics of Sugars

**Figure 5:**
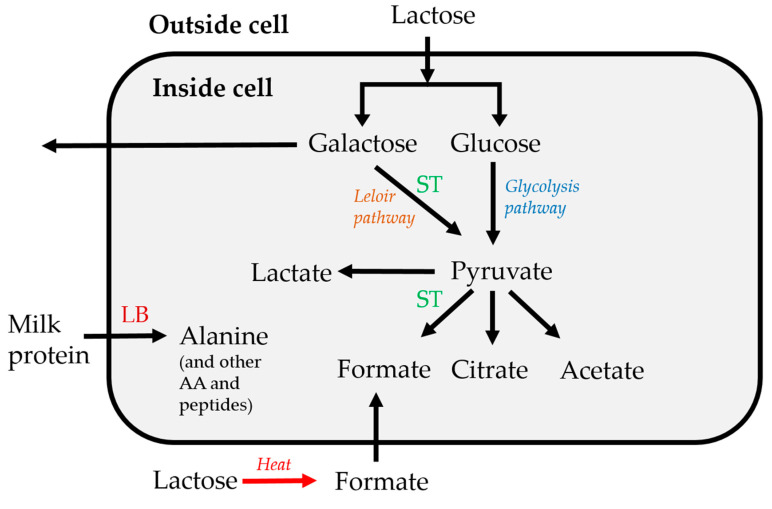
Metabolic relations between the main metabolites found in this study. AA = amino acids.

Lactose is transported into the cell similarly for both ST and LB, just like the transportation of glucose outside the cells [19]. Lactose is then cleaved to glucose and galactose by a cytoplasmatic ß-galactosidase for both species. After this, galactose is excreted out of the cell, whereas glucose is employed in the glycolysis pathway [19]. Some strains of ST can ferment galactose: galactose is taken up by ST and used in the Leloir pathway to produce lactate and CO_2_ (Figure 5). This is especially the case for strains of ST that lack urease activity and cannot produce CO_2_ that way [20]. LB, on the other hand, does not have the genes for galactose metabolism, thus it can only use lactose and glucose. Moreover, some strains with no possibility to transport glucose can just catabolize intracellular glucose released by lactose metabolism. Therefore, both species can produce and secrete glucose, so it is one of the nutrients that they can share [19].

In the present study, lactose shows different behaviors depending on the heat treatment of the milk. In LT heated milk, MX samples exhibit the largest consumption after 24 h, indicating a protocooperation between the two strains. In the HT heated milk, LB has the largest lactose consumption. In this case, formate is formed by conversion of lactose during the heat treatment (Figure 5), and therefore, the formate produced by ST is redundant [15].

Lactose metabolism generates primarily glucose and galactose. The signals of glucose, though, are largely overlapping with signals from other sugars, lactose in particular. This signal overlapping could cause the uneven and peculiar kinetic behavior found in the lactose kinetics plot (Figure 2).

Galactose, instead, has clearer NMR signals, which can give information on its metabolism. When using LT milk, galactose has the greatest increase in concentration in MX starter due to protocooperation, whilst no difference in its development is observed between LB and MX in HT milk, since LB uses the formate produced by the heat treatment. Galactose is formed by decomposition of lactose and generally not used as substrate by the LAB, particularly not by LB, so its increase is linearly proportional to lactose decrease [15].

Galactose-like signals, as well as glucose-like signals, could also arise from exopolysaccharide (EPS) produced by the lactic acid bacteria. These EPS can have different forms (orbs, capsule, link to bacterial cell or not), but their composition largely consists of the aforementioned sugars and rhamnose. Most strains of LB and ST can produce ropy EPS, a property used to improve rheological quality in yoghurt [15]. Therefore, the presence of galactose could also be an indication of these characteristics in the product.

The trends in these sugar metabolites, and lactate, are quite similar to the ones tested by Sørensen et al. [19], who quantified these molecules in fermentations using mutant strains of ST, LB and their combinations. Their mutants were consuming a substantially larger amount of lactose and metabolizing some galactose and secreting the rest, together with glucose, back into the milk, producing yoghurt with very low lactose and a greater sweetness. This shows that monitoring fermentation as proposed in the present study can allow understanding of the composition of sugars and its fine-tuning to fit companies and consumers’ needs.

### 3.2. Fermentation Kinetics in Deprotonated Organic Acids and Other Molecules

Acetate is a product in the metabolism of pyruvate, but it is also part of citrate metabolism. After 8 h of fermentation, in both milk types, LB has the highest production of acetate (data not shown), then it decreases at 24 h, especially for the HT milk.

Citrate displays a very similar development across cultures and temperatures: with very little to no development in the first 4 h, a dramatic increase between 4 and 5 h and then again little to no development. It has been shown that strains of ST and LB cannot metabolize this molecule, therefore, pyruvate is instead used, from sugar metabolism, to produce diacetyl and acetoin, two important aroma molecules for yoghurt [15].

The spectral region where formate is found is affected by the presence of broad signals especially at lower time-points of fermentation. This generates uncertainty in the estimation and significant variation among replicates and samples. Some trends, though, can still be observed. At the beginning of fermentation, as expected, there is a greater concentration of formate in HT milk, since lactose is decomposed into formate during intense heat treatment [15]. For what concerns its development and kinetics, it decreases when LB is used as starter, since LB cannot produce formate and instead employs it for growth. With ST, formate can be produced from pyruvate [15]. In MX, there is a slight decrease between 7 and 8 h of fermentation, indicating that LB is using it faster than ST can produce it.

Pyruvate is found in higher concentrations in MX and ST, with MX showing a decrease in pyruvate concentration after 8 h, due to its employment by LB. Pyruvate is mainly produced by ST. ST has a pyruvate-formate lyase, which LB lacks, therefore, ST can provide LB with the necessary formate for purine biosynthesis and for growth [21]. Moreover, pyruvate can be used to produce citrate [22], and through citrate metabolism it is also used by ST to produce acetate, formate, acetaldehyde and diacetyl, and it is thus very important in the aroma development of yoghurt [20]. The rapid decrease in pyruvate in samples containing LB, however, is probably caused by its employment for lactate production [23], also mirrored by the lactate increase in these samples at the same time-points.

Fumarate, another deprotonated organic acid, shows a great increase in the first 5 h when ST are present, then it is rapidly consumed in MX samples. In fact, fumarate produced by ST has been shown to stimulate LB [24]. Succinate shows a different behavior, with LB having the greater concentration (data not shown). This was also found in previous studies [25,26]. It was speculated that heat treatment could generate some compounds that alter the carbohydrate metabolism of LB, causing this production of succinate [26]. Lactate shows an increase in concentration during fermentation with a faster slope after 4 h and a further increase in concentration from 8 to 24 h. ST is the strain that displays the lowest increase in lactate and the highest is observed for LB and MX in HT milk. Lactate is also produced through pyruvate [14,22,27], thus, the decrease in pyruvate for LB and MX can also be explained by lactate production.

1,3-dihydroxyacetone is an intermediate in sugar metabolism [28]. It was previously found in greater concentrations in a mix culture of ST and LB and in pure ST culture by Settachaimongkon and colleagues [25]. In this case it is produced only at later fermentation stages, and particularly for LB. This might be due to the fact that ST can employ galactose through the Leloir pathway, whilst LB can only metabolize glucose.

Alanine is produced via proteolysis mostly by LB and MX, in higher concentrations in the latter case, possibly due to protocooperation. ST can also proteolyze proteins to alanine, but there is very little to no increase between the last two time-points. ST in fact lacks proteases in the bacterial wall and thus has low proteolytic activity [20]. The proteolytic activity of LB is also particularly evident in the case of aromatic amino acids such as phenylalanine. Previous studies have shown that ST does not produce amino acids such as tyrosine or phenylalanine but employs them when produced by LB [29,30].

In this case, phenylalanine is also greatly increased in LT milk. The heat treatment could induce polymerizations from crosslinking such as from the Maillard reaction [31]. In this way free amino groups could be less available [32]. For what concerns proline, the corresponding interval appears to decrease in time, to then increase again after 5 h for LB and MX samples. By visual inspection of the spectra, though, it is possible to understand that the initial value is caused by the presence of larger peptides and proteins, whilst the actual signal from proline arises only at later stages in LB and MX samples, as displayed by the kinetic pattern after 5 h. This is why it is important to use spectral visualization in parallel with statistical analysis. The sole use of spectral areas as investigation data can be misleading, especially in complex regions like this one. The production of proline by LB and not by ST was also documented by Beshkova et al. [29].

3-hydroxyisobutyrate was also found at higher concentrations in LB and MX samples, especially in HT milk. This metabolite derives from the breakdown of valine. Therefore, the proteolytic activity of LB can be the cause of the difference between ST and LB for this metabolite.

### 3.3. The Investigation of Fermentation through Application of NMR and Chemometrics

The ^1^H-NMR spectra obtained for this study proved in many cases to be affected by field inhomogeneities across the fermentations. The intrinsic characteristics of the milk sample undergoing fermentation change quite drastically both from a chemical point of view but also from a physical point of view: specifically, the viscosity is increased due to the synthesis of exopolysaccharides and creation of casein networks [33]. Even when quenched and resolubilized, some thickening effect remains that deteriorates the NMR spectra. In fact, this can cause the presence of broader signals even for smaller molecules, hindering the comparison of specific concentrations. This change in viscosity is also what caused different spectral quality results than the ones obtained from a previous investigation, which instead employed a chemically defined interaction medium [14]. Future studies should take a closer look at this problem and investigate the different sample extraction and preparation steps, for example those described by Settachaimongkon et al. [25]. A more fundamental but much more involved solution would be to employ a hyphenated method such as LC-NMR [34,35].

The post-acquisition pipeline, instead, proved to be a good example for investigating fermentation in milk and possibly for similar studies. The application of interval selection proved to be a powerful tool to focus on the relevant information and discard noisy data. SigMa software [36] was used in this study to identify and quantify the molecules present in the samples and will possibly in the future contain a specific library for foods such as milk. This can help in future studies to have a more robust picture of the kinetics of determined molecules of interest.

In this study, it was possible to investigate the kinetics of 37 selected spectral regions, of which 25 belonged to 19 identified molecules, and check how these were impacted by the different strains and heat treatments. Moreover, how the strain and initial matrix could affect the composition of the final yoghurt product was assessed. It is to be noted that it is important to use a combination of spectral visual investigation and statistical analysis to assess the changes in the sample matrix, due to the complex changes occurring in the product during fermentation. In fact, the changes experienced in the aliphatic and aromatic region, for example, cannot be fully understood by a simple glance at their kinetics. The total area in these spectral regions seems to decrease at first, to increase again at later fermentation stages. In reality, the matrix changes drastically, going from large molecules such as proteins and peptides to small amino acids, and this is particularly important to understand for the nutritional characterization of the final product.

With this in mind, the described pipeline can be employed in further investigations and similar studies, as it has proven to give a good insight into the protocooperation of LAB strains and the impact of a greater heat treatment in the initial matrix. This is particularly useful for companies producing strains, but also for dairy companies, in order to know exactly which strains they need and what is the best initial substrate matrix in order to obtain the desired characteristics of their yoghurt.

This study constitutes a starting point to develop a specific analytical pipeline for the investigation of fermentation of milk by employing ^1^H-NMR spectroscopy. This platform can give insight into the metabolic processes happening in milk during fermentation and help understanding of the complex mechanism regulating the employment and production of specific molecules by the starter cultures. Moreover, thanks to its relative simplicity in sample preparation and fast spectral acquisition, it is the ideal platform for investigations of fermentations.

## 4. Materials and Methods

### 4.1. Starter Cultures and Media

Two different types of milk were used in the study. Both types were supplied by a commercial supplier and produced from the same skim milk powder batch. The only difference between milks was the heat treatment temperature used for sterilization. The first type was heat treated at 105 °C for 30 min (HT), while the second type was treated at 99 °C for 30 min (LT). Samples displayed initial differences immediately using visual inspection, with HT milk appearing a more yellow color, whilst LT milk was white. The development of the yellow color might be due to Maillard reactions: the heating of lactose and amino acids/proteins could in fact produce brown compounds [15]. A slight pH difference was present at T0 with HT having a pH value of 6.61 ± 0.01 and LT of 6.68 ± 0.05.

**Figure 6:**
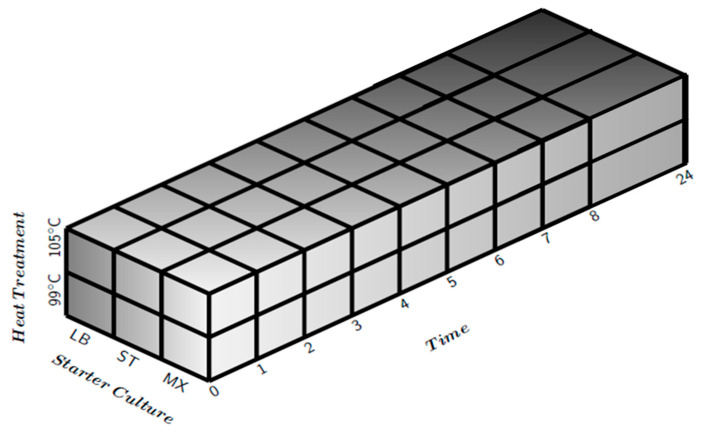
Illustration of the experimental design: it is a three-way factorial design with two different heat treatments (105 °C and 99 °C), three different starter cultures (LB, ST and MX) and ten different fermentation times (0, 1, 2, 3, 4, 5, 6, 7, 8 and 24 h). All measurements were duplicated.

Three different freeze-dried starter cultures were provided by a commercial supplier. The starter cultures used were *Streptococus thermophilus* (“ST”), *Lactobacillus delbrueckii* ssp. *bulgaricus* (“LB”) and a combination of the two (“MX”). The experimental design is outlined in Figure 6.

### 4.2. Preparation of Milk Samples for Fermentation

The freeze-dried cultures, in either an amount of 2 or 4 g, were diluted with 200 mL of milliQ water. This solution was then stomachered for 2 min, before being diluted into the milk sample.

Milk was heated in a water bath to either 37 °C or 43 °C, according to the starter culture to be added. Table 1 shows the temperatures and inoculations rates for the different samples.

An amount of 40 mL of inoculated milk was transferred into 40 mL plastic falcon tubes and closed with a lid. These falcons were then transferred to the respective water bath. Two falcons (as replicates) were sampled for each milk every hour in the first 8 h and then finally after 24 h, i.e., for each combination of starter and milk heating temperature there were a total of 20 × 40 mL plastic falcon tubes.

Before pH measurement, samples were cooled in an ice bath and then measurements were performed as soon as possible. For NMR, 6 mL of milk were collected into a falcon.

### 4.3. Chemicals

Chemicals and reagents. All chemicals and reagents used in this study were purchased from Sigma-Aldrich (Søborg, Denmark) unless otherwise stated. These included deuterium oxide (D_2_O, 99.9 atom % D), sodium phosphate monobasic monohydrate (NaH_2_PO3, H_2_O), sodium phosphate dibasic heptahydrate (Na_2_HPO_3_, 7 H_2_O), sodium salt of 3-(Trimethylsilyl) propionic-2,2,3,3-d4 acid (TSP, 98 atom % D, ≥98.0%) and sodium azide (NaN3, ≥99.5%), used for quenching and sample stability. The Milli-Q water used throughout the study was purified using a Millipore lab water system (Merck KGaA, Darmstadt, Germany) equipped with a 0.22 μm filter membrane.

### 4.4. Preparation of NMR Samples

NMR samples were centrifuged for 10 min at 3500 rpm and 4 °C. An aliquot of 350 μL of supernatant (when present, otherwise of sample) was taken from the centrifuged sample and mixed with 350 μL of buffer in a cryovial. The 0.3 M sodium phosphate buffer (pH = 6.47) was prepared using a modified version of the protocol employed by Ebrahimi et al. [14]. Deuterium oxide (D_2_O, 99.9 atom % D) (20%) was added to the abovementioned buffer solution together with 25 mg of sodium azide (NaN_3_) and 7 mg of the reference compound TSP. Samples were then snap-frozen in liquid nitrogen and kept at −80 °C until NMR analysis.

### 4.5. NMR Analysis

Samples were thawed at room temperature for 30 min. An aliquot of 600 μL was transferred into a 5 mm (o.d.) NMR tube for analysis. ^1^H-NMR measurements were performed on a Bruker Advance DRX-500 Spectrometer (Bruker Biospin, Rheinstetten, Germany), operating at a Larmor frequency of 500.13 MHz for ^1^H and equipped with a BACS-60 sample changer (Bruker Biospin, Rheinstetten, Germany). Automation of the overall measurement procedure was controlled using an Icon NMR (Bruker Biospin, Rhein-stetten, Germany).

The pulse sequence *zgcppr* was employed, with a pre-saturation followed by a composite 90-degree pulse, a recycle delay of 5 s, 64 scans, 10,000 Hz of spectral width and an acquisition time of 1.639 s.

Spectra were automatically phased and baseline corrected using the Bruker software Topspin 3.5 (Bruker Biospin, Rheinstetten, Germany) and then imported into MatLab (The Mathworks Inc., Natick, MA, USA). Five sample measurements were identified as erroneous by visual inspection of the spectra and were removed for further analysis.

Spectra, after normalization to receiver gain, were then processed using SigMa software [36]. They were initially referenced to the TSP signal at 0.0 ppm, then pre-aligned using large spectral regions and employing the icoshift algorithm [37]. Baseline drifts were also corrected through SigMa.

A total of 37 individual peak areas were identified using SigMa. These included 25 signature signals (SS) from 19 molecules, 3 signals of unknown spin systems (SUS) (unknown—not assigned) and 9 bins (complex intervals representing many overlapped and/or shifted signals) (Table A1 in Appendix A). These intervals were quantified and the obtained metabolite table was used for further analysis.

### 4.6. Statistical Analysis

Principal component analysis was performed on autoscaled peak areas in R using base R functions [38].

Heatmap hierarchical clustering analysis was carried out on the peak areas of the 37 selected intervals. To measure distance among samples, Pearson’s correlation coefficient was used, in order to focus on the pattern of the samples’ concentration profiles rather than their magnitudes. To link groups of samples, average linkage was used [17]. To choose the number of non-random clusters, the gap statistic was used, by choosing the number of clusters as the smallest number of k clusters such that the gap statistic is within one standard deviation of the gap at k + 1: gap(k) ≥ gap(k + 1) − s_k+1_ [18]. Hierarchical clustering was carried out in R using base R functions [38]. Choosing the number of clusters using the gap statistic was done using the factoextra package [39]. Visualization of heatmap hierarchical clustering was carried out using the R package pheatmap [40]. All plots from statistical analyses were made using the R package ggplot2 [41].

## 5. Conclusions

This study has shown a metabolomics approach for the investigation of milk fermentation for yoghurt production employing different starter cultures and different heat treatments on the initial matrix. Through this approach, mimicking more real-life conditions, it is possible to understand the behavior of starter cultures when actually employed in a milk sample, as used by yoghurt producing companies. In this way, more information on the actual impact of protocooperation on the final product was achieved; in fact, it was possible to see how some metabolites such as formate or fumarate can be produced by ST and employed by LB, and vice versa for amino acids. Moreover, the employment of a higher temperature treatment for the milk matrix generated formate, and therefore reduced the necessity of ST for LB growth stimulation. This information can be particularly useful for food companies and is not always extractable from studies employing starter growth media which do not resemble a real-life food matrix. The drawback of the present approach is the complexity of the sample matrix and the difficulty in comparing spectral areas when large molecules such as proteins are transformed into small amino acids. Moreover, in some of the samples in the present study the quality of the NMR spectra were negatively affected by the viscosity changes during the fermentations. This problem was overarching in a previous study on intact yoghurt samples [14] but still remained a problem even though the yoghurt samples were spun down and resolubilized. If this problem should be completely eliminated an improved analytical protocol is needed in which the large molecule compounds increasing sample viscosity are separated from the small molecules.

## Figures and Tables

**Table 1:** Inoculation concentrations and fermentation details.

Strain	Temperature (°C)	Inoculation %	1st Dilution (g)	2nd Dilution (g)	Analysis Time (h)
*Streptococcus thermophilus*	37	0.00172	4.00	0.94	24
*Lactobacillus delbrueckii* ssp. *bulgaricus*	43	0.00050	2.00	0.54	24
*Streptococcus thermophilus* and *Lactobacillus delbrueckii* ssp. *bulgaricus*	37	0.00340	4.00	1.86	24

## Appendix A

**Table A1:** Spectral regions identified and relative ppm range. Unassigned signals (SUS) and BINS are also reported.

Interval Name	End ppm	Initial ppm
Valine	1.07	1.03
3-hydroxyisobutyrate	1.22	1.18
Lactate	1.36	1.30
Threonine	1.40	1.36
Alanine	1.48	1.45
Acetate	1.95	1.90
N_acetyl_derived_AA_and_methionine	2.08	2.03
Pyruvate	2.39	2.38
Succinate	2.45	2.41
Citrate1	2.60	2.51
Citrate2	2.74	2.64
Lactose	3.34	3.27
Galactose	3.52	3.47
Galactose	4.10	4.07
Lactate2	4.19	4.11
1,3-dihydroxyacetone	4.43	4.42
Lactose	4.49	4.43
Galactose	4.62	4.56
Lactose_glucose	4.71	4.64
Anomeric	5.31	5.20
Fumarate	6.55	6.51
Tyrosine_else	7.21	7.17
Phenylalanine	7.46	7.41
Hippurate	7.87	7.81
Formate	8.48	8.44
Unk_singlet_acetone * (SUS)	2.24	2.22
Unknown_singlet3 (SUS)	6.22	6.18
Unknown_q (SUS)	9.71	9.64
Aliphatic_region (BIN)	1.00	0.83
Proline (BIN)	2.03	1.95
Sugars (BIN)	3.90	3.53
Glycolate_galactose_lactose (BIN)	4.02	3.90
Galactose_lactate (BIN)	4.11	4.10
Aromatic1_tyrosine (BIN)	7.04	6.78
Aromatic2_phenolics (BIN)	7.41	7.21
Aromatic3_hyppurate (BIN)	7.77	7.51
Aromatic4_adeninesinglets (BIN)	8.40	7.94

* This signal corresponds in shape and chemical shift to acetone but could only be tentatively assigned.
